# Supplementary material for: Health literacy and associated factors among undergraduates: A university-based cross-sectional study in Nepal
Source: PLOS Glob Public Health. 2021 Nov 8;1(11):e0000016. doi: 10.1371/journal.pgph.0000016 (PMC10022320; doi:10.1371/journal.pgph.0000016)
Supplement: S1 Tool — (DOCX) [file pgph.0000016.s002.docx]

**Health Literacy Assessment (HLS-EU-Q 16)**

| **Questions about how it is for you to find, understand and use information related to health, illness and medical care.** | | | | | |
| --- | --- | --- | --- | --- | --- |
| *Select the option on each line that best matches your answer.* | | | | | |
|  | Very easy | Easy | Difficult | Very difficult | Don’t know |
| 1. How easy/difficult is it for you to find information on treatments of illnesses that concern you? |  |  |  |  |  |
| 2. How easy/difficult is it for you to find out where to get professional help when you are ill (e.g. doctor, pharma­cist or psychologist)? |  |  |  |  |  |
| 3. How easy/difficult is it for you to understand what your doctor says to you? |  |  |  |  |  |
| 4. How easy/difficult is it for you to understand your doc­tor´s or pharmacist´s instruction on how to take a pre­scribed medicine? |  |  |  |  |  |
| 5. How easy/difficult is it for you to judge when you need to get a second opinion from another doctor? |  |  |  |  |  |
| 6. How easy/difficult is it for you to use information the doctor gives you to make decisions about your illness? |  |  |  |  |  |
| 7. How easy/difficult is it for you to follow instructions from your doctor or pharmacist? |  |  |  |  |  |
| 8. How easy/difficult is it for you to find information on how to manage mental health problems such as stress and depression? |  |  |  |  |  |
| 9. How easy/difficult is it for you to understand warn­ings about behaviour (e.g. smoking, low physical activity and drinking too much)? |  |  |  |  |  |
| 10. How easy/difficult is it for you to understand why you need health screenings (such as breast exam, blood sugar- or blood pressure test)? |  |  |  |  |  |
| 11. How easy/difficult is it for you to judge if the informa­tion on health risks in the media is reliable (e.g. from TV or internet)? |  |  |  |  |  |
| 12. How easy/difficult is it for you to decide how you can protect yourself from illness based on information in media (e.g. newspapers, leaflets and internet)? |  |  |  |  |  |
|  |  |  |  |  |  |
| *Select the option on each line that best matches your answer.* | | | | | |
|  | Very easy | Easy | Difficult | Very difficult | Don’t know |
| 13. How easy/difficult is it for you to find out about activi­ties that are good for your mental well-being (e.g. medi­tation, exercise and walking)? |  |  |  |  |  |
| 14. How easy/difficult is it for you to understand advice on health from your family members or friends? |  |  |  |  |  |
| 15. How easy/difficult is it for you to understand informa­tion in the media on how to get healthier (e.g. from the internet, daily or weekly magazines)? |  |  |  |  |  |
| 16. How easy/difficult is it for you to judge which everyday behaviour is related to your health (e.g. eating habits, exercise habits and drinking habits)? |  |  |  |  |  |
