# Supplementary material for: Health literacy and associated factors among undergraduates: A university-based cross-sectional study in Nepal
Source: PLOS Glob Public Health. 2021 Nov 8;1(11):e0000016. doi: 10.1371/journal.pgph.0000016 (PMC10022320; doi:10.1371/journal.pgph.0000016)
Supplement: Attachment — Submitted filename: Response to Reviewers 9.9.21.docx [file pgph.0000016.s003.docx]

**Reviewers’ reports:**

**Reviewer #1**

Methods:  **1. Method is well written and explained the tools adequately. But a brief description of the web-based survey tool, the language in which it was delivered would be welcome. Did all the tools [health literacy scale, financial, self-esteem and health status] were put in the online survey tool, and whether it was delivered in English or Nepali Language may help the readers to understand the data collection process. A line or two on the consent process should be considered.**

Thank you for your suggestion. We have mentioned the details on consent taking process. This can be now read as below in the manuscript.

*“English version of the European Health Literacy Survey Questionnaire with 16 items (HLS-EU-Q16) was used to assess health literacy.”(Page number- 5, Line numbers- 124,125, Measurements)*

*“All the respondents were informed about the aims and objectives of the study by including the written consent form in the questionnaire itself.” (Page number- 7, Line numbers- 172-174, Ethical consideration)*

**2. A brief description of the self-reported financial scale or how the information was collected will give the readers better insight into the analysis. Otherwise, attachment tools for financial status or health status can be considered as supplementary documents as per the journal guidelines can be considered.**

Thank you for your suggestion. We used the self-reported financial scale that was already used in a study conducted among university students in Ghana (<https://pubmed.ncbi.nlm.nih.gov/31637363/>). We have elaborated it and cited the reference article in the measurements section as below;

*“Self-rated financial status was measured on a scale of 1 to 6 which included the responses from very poor to very rich (17), categorized as a new variable as: 1–2 (poor) and 3–6 (good).”(Page number- 6, Line numbers- 155-157, Measurements)*

Results:

**3. Table 2: Table 2 has been described as, 'When comparing all competencies over the domains of healthcare, disease prevention, and health promotion, the mean score per item was highest within the domain of health promotion [Mean (SD): 2.86 (0.55)] and lowest in the domain of disease prevention [Mean (SD): 2.86 (0.63)]. But in table 2, the Mean SD of the disease prevention is 2.74 and not 2.86. The authors should check this discrepancy.**

Thank you for pointing the error in our manuscript. Now the discrepancy is solved. Actual value is added.

Discussion:
**4. The paragraph above the strength and limitation where the authors refer to similarity with a study in Denmark [Ref 18]. It would be better if the authors briefly describe the similarity that they are trying to point.**

Thank you for your suggestion. Now we have rewritten the sentences as below in the manuscript.

*“In our study, the mean score per item (over all competencies) was lowest for appraising health information and while comparing all competencies over the three domains, the mean score per item was highest within the domain of health promotion. Therefore, our findings corroborate with findings from study conducted in Denmark.”(Page number- 14, Line numbers- 263-266, Discussion)*

Generic
**5. The lack of page numbers and line numbers is a bit problematic for reviewing.**

We apologize for this error that made it difficult for reviewing the manuscript.

**Reviewer #2**

Topic
**1. Authors could make the topic more specific. Since, this study has been done in only one university of Nepal, it would not be fair to say the study among the university students. The current topic gives an idea to the readers that a study is among the university students in Nepal which, in fact, is not.**

Thank you for your suggestion. Acknowledging the reviewer’s comment we have revised the topic accordingly.

*“Health Literacy and Associated Factors among Undergraduates: A University Based Cross-Sectional Study in Nepal” (Page number 1, Title page)*

Abstract
**1. The author has used HLS-EC-Q16 as a key word, but I did not find this abbreviation used anywhere in the abstract. I would suggest using the abbreviation first, at least once, and then use it in the keyword section.**

Thank you for the suggestion, it has been done accordingly.

*“The 16-item short version of the European Health Literacy Survey Questionnaire (HLS-EU-Q16) was used to measure students’ health literacy levels.” (Page number 2, Line number- 28, Abstract)*

Introduction

**1. Not enough literature review has been done. How can limited health literacy leads to the economical burden and health inequalities in the society, you need to support this argument with evidence since you have mentioned this twice (in the abstract and second paragraph of an introduction).**

Thank you for the suggestion, now the relevant articles are cited to support the statement. The articles added are in reference number 5 and 6.

*Reference 5:* [*https://hsrc.himmelfarb.gwu.edu/sphhs_policy_facpubs/172/*](https://hsrc.himmelfarb.gwu.edu/sphhs_policy_facpubs/172/)

*Reference 6:* [*https://pubmed.ncbi.nlm.nih.gov/15610334/*](https://pubmed.ncbi.nlm.nih.gov/15610334/)

**2. The question like why this study was done in Nepal or how this study is relevant to Nepalese context is crucial. What are those believes, practices and mis-conception prevalent is Nepalese society which are preventing people to get informed about health information or have implication on health and disease in Nepal. Why was it so important to conduct this study in Nepal? Why you choose undergraduate student in Nepal? How this group (undergraduate) is so important in the Nepalese context that compel you to conduct this study? For example, did you choose this study group because this group represents the larger fraction of Nepalese population or because this group can influence the larger percentage of Nepalese population or because of something else? You need to work more on this. By only saying that undergraduate is a transition period, and it is always misunderstood as an educated group is enough to conduct this study. I think, enough studies need to be examined to answer these questions. The relation between three: Health Literacy, Nepal and Undergraduate students should be strong to carry out this study. This convinces others as well to make another study, expand this work and to contribute more to the area. When your rational to conduct a study in any region is strong, being an international reader who do not know about Nepal, could understand your work more clarity and easily correlate the situation. It also makes an international reader to read a paper with the eyes of clarity.**

Thank you for this suggestion. The relevant evidences are added to support the statements and rationale behind selection of undergraduates is expanded in the manuscript as below;

*“Understanding the health literacy levels of these younger populations and then addressing any gaps provides a mechanism towards producing health literate professionals who are able to understand and respond to the health literacy needs of the families and communities (14,15). This population constitutes a major proportion and is crucial for the success of any health related promotion or prevention efforts. They are very receptive to information, and therefore, healthy behavior established at this phase of life is more likely to be continued (16).”(Page number- 3 and 4, Line numbers- 71-77, Introduction)*

**3. In the 4th paragraph, the author has aimed to assess the level of comprehensive health literacy through this study. What does the word “comprehensive” means here? Does this questionnaire claim to measure the level of comprehensive health literacy? If yes, please state the word comprehensive with the evidence from claim.**

Thank you for the suggestion. We have removed the word “comprehensive” from our manuscript.

Methodology:

**1. I think, the author has not explained the inclusion and exclusion criteria very well which have created a confusion of understanding. The authors say that all undergraduates’ students are included. But I guess there must be international students in IoM, IoE or other institute pursuing their undergraduate degree. What about them? Did you include them or not? If yes, do they represent Nepal? Please make this clear.**

Thank you for the suggestion. International students were excluded from our study. Now we have revised our inclusion and exclusion criteria.

*“Postgraduate students, international students, and students undertaking affiliated programs were excluded.” (Page number- 5, Line number- 103,104, Study population)*

**2. The author has excluded the postgraduates’ students and affiliated programs. But what about the constituent campuses in IoM. There are 7 constituents’ campuses and 1 central department of public health under the IoM. Did you include the students from those constituents’ campuses? Are there any other constituent campuses under other institutes like the Institute of Medicine have? You should mention these thoughts very clearly. So that the readers should not have any confusion.**

Thank you for asking this queries and providing us a chance to clarify and strengthen the methods section of our manuscript. Now we have clearly defined the study setting and this can be now read as below in the manuscript.

*“All the five institutes of the university; Institute of Medicine (IOM), Institute of Engineering (IOE), Institute of Forestry (IOF), Institute of Agriculture and Animal Science (IAAS), and Institute of Science and Technology (IOST), were selected as the study sites. Then, one campus from each of the institutes was purposively selected. Maharajgunj Medical Campus, Pulchowk Campus, Hetauda campus, Lamjung Campus, and Trichandra Multiple Campus were selected respectively from IOM, IOE, IOF, IAAS, and IOST to recruit the participants.” These campuses were selected as these are the pioneer campuses under the respective institutes of Tribhuvan University. (Page number- 4, Line numbers- 90-95, Study setting)*

**3. The author has collected the sample from each institute proportionally based on the size of the population of each institute. So, what is the number of population/students enrolled in each institute in the corresponding year? How do I check whether they have collected the responses proportionally or not? How do I validate your sample taken from each institute? You must include the total number of students enrolled in each institute in the year in which study was pursued.**

Thank you for asking this question and providing us a chance to correct our error.

We proposed to conduct this study by taking proportionate samples from all the five Institute of Tribhuvan University based on the population size of each institute. We were determined to collect the responses by conducting face to face interview with the students at their own campus. However, we weren’t able to conduct this study as per previously designed manner and had to proceed with online survey due to the COVID-19 pandemic that forced the country to implement strict lockdown, travel restriction, and closure of academic institutions. We have mentioned this in our limitations.

Now we have corrected our sampling procedure on the manuscript and added it on limitations of our study as well. We have included the number of participants from each institute in the Table 1.

*“Responses were collected from each of the five campuses of respective institutes. The convenience sampling technique was employed to select participants.”(Page number- 5, Line numbers- 109-111, Study design and sample)*

*“Further studies with better sampling procedure using a longer version of health literacy survey questionnaire and qualitative methods to explore more on determinants of health literacy are recommended.” (Page- 15, Line numbers- 288-290, Conclusion)*

**4. Did you translate the questionnaire into your local Nepalese language? If yes, how did you do that? Is the questionnaire tested before in the local language or the translated questionnaire have been piloted before? If you did not translate the questionnaire, did you receive any difficulties from the participants about the understanding of the words used in the questionnaire? Please make this very clear. This is important in many senses. One would be, it may give an idea to other researchers who wants to replicate or expand your work in the future by using the same questionnaire in Nepal.**

English version of the Health Literacy Questionnaire (HLS-EU-Q16) was administered to the participants. We have added this on revised manuscript. During the pretesting no one reported the difficulties in understanding the questionnaire and we decided to administer questionnaire in English version. We have explained this in the methodology.

This study tools was adapted from previous studies conducted in similar study population in Ghana. (<https://pubmed.ncbi.nlm.nih.gov/31637363/>)

**5. The author has used a scale of 1 to 6 to measure self-rated financial status. Could you please clarify how did you build this scale? Is this an international standard? Is there any formula to develop this scale?**

Thank you for your suggestion. We used the self-reported financial scale that was already used in a study conducted among university students in Ghana (<https://pubmed.ncbi.nlm.nih.gov/31637363/>). We have elaborated it and cited the reference article in the measurements section.

*“Self-rated financial status was measured on a scale of 1 to 6 which included the responses from very poor to very rich (17), categorized as a new variable as: 1–2 (poor) and 3–6 (good).”(Page number- 7, Line numbers- 155-157, Measurements)*

**6. The author says limited access to the internet may have discouraged the students from participating in the survey. Does any student refused/denied participating in this survey due to internet problem or any other problem? If yes, what was the reason of denial. Was it internet or any cultural believes or any other reason? You should provide information regarding the refusal of participants if there was any.**

Our estimated sample size was 475, of which we included 469 in the final analysis as 6 responses were incomplete. We assumed that the 6 incomplete responses might be due to the problem related to internet considering the fact that everyone may not have access to Wi-Fi or quality internet as it requires reasonable access to internet to fill out Google form questionnaire.

**7. Why did the author used shorter version of HLS-EC questionnaire? Why not the longer version of this questionnaire (HLS-EC Q16 vs Q47)?)? Please explain this. This may help you to identify strength or weakness of this study.**

Thank you for flagging the importance of longer version of the questionnaire. However, many studies have conducted using a short version of the HLS-EU-Q16;

[*https://bmcpublichealth.biomedcentral.com/articles/10.1186/s12889-020-08498-8*](https://bmcpublichealth.biomedcentral.com/articles/10.1186/s12889-020-08498-8)

[*https://www.mdpi.com/1660-4601/17/5/1683*](https://www.mdpi.com/1660-4601/17/5/1683)

[*https://pubmed.ncbi.nlm.nih.gov/31637363/*](https://pubmed.ncbi.nlm.nih.gov/31637363/)

Considering we had to collect responses through Google forms due to the current pandemic and from the practicability point of view (relatively easy to administer and less questions) we used the 16 item short version of the HLS-EU questionnaire. We have included this on conclusion section and future research using longer version of questionnaire are recommended.

Results
**1. The author in the Table 3 has categorized an academic year into Higher year and Lower year. What does higher and lower year mean? How did you classify this? Which year of students are included in higher and which groups are kept in lower year? Please state this.**

Thank you for your suggestion. Now we have clarified this on measurement section.

*“Academic year of the students was categorized into “lower year” and “higher year’, where 1^st^ or 2^nd^ year of the study was regarded as lower year and ≥3^rd^ year as higher year.”(Page number- 6, Line numbers- 151-153)*

**2. Be careful about the correctness while writing the numbers and decimals. For example, you have used two values of mean (SD): 2.86 and 2.74 for disease prevention domain. Which one is correct? Please check the overall manuscript for such minor errors to convey the right idea.**

Thank you for pointing the error in our manuscript. Now the discrepancy is solved. Actual value is added.

Discussion and conclusion (Strengths and limitations)

**1. There is huge difference in the sample size between health and non-health related students (26% vs 74%). Based on this sample difference, how can you say that the health-related students demonstrate higher level of health literacy? What if that was because of lower sample size of health-related students?**

Thank you for suggesting us to further elaborate the limitations section of our manuscript about it. 4 out of 5 institutes were non-health related, that led to higher number of participants from non-health related institutes. This is now mentioned in the discussion of the limitations of our study.

*“As most of the participants were from non-health background, which might have created the discrepancy while analyzing the health literacy level in terms of health-related students versus non-health related students” (Page number- 14, Line numbers- 276-278)*
